# The new antioxidant 1-benzoyl-6-hydroxy-2,2,4-trimethyl-1,2-dihydroquinoline has a protective effect against carbon tetrachloride-induced hepatic injury in rats

**DOI:** 10.7555/JBR.36.20220098

**Published:** 2022-07-28

**Authors:** Evgenii Dmitrievich Kryl'skii, Darya Andreevna Sinitsyna, Tatyana Nikolaevna Popova, Khidmet Safarovich Shikhaliev, Svetlana Mikhajlovna Medvedeva, Larisa Vladimirovna Matasova, Valentina Olegovna Mittova

**Affiliations:** 1 Department of Medical Biochemistry and Microbiology, Voronezh State University, Voronezh, Voronezh region 394018, Russia; 2 Department of Organic Chemistry, Voronezh State University, Voronezh, Voronezh region 394018, Russia; 3 Department of Clinical Laboratory Diagnostics, Voronezh State Medical University named after N.N. Burdenko, Voronezh, Voronezh region 394036, Russia

**Keywords:** CCl _4_-induced hepatic injury, oxidative stress, 1-benzoyl-6-hydroxy-2,2,4-trimethyl-1,2-dihydroquinoline, antioxidants

## Abstract

Liver diseases with the central pathogenetic mechanism of oxidative stress are one of the main causes of mortality worldwide. Therefore, dihydroquinoline derivatives, which are precursors of hepatoprotectors and have antioxidant activity, are of interest. We have previously found that some compounds in this class have the ability to normalize redox homeostasis under experimental conditions. Here, we initially analyzed the hepatoprotective potential of the dihydroquinoline derivative 1-benzoyl-6-hydroxy-2,2,4-trimethyl-1,2-dihydroquinoline (BHDQ) for carbon tetrachloride (CCl _4_)-induced liver injury in rats. Results suggested that BHDQ normalized the alanine aminotransferase, aspartate aminotransferase, and gamma-glutamyl transpeptidase in serum. We also observed an improvement in liver tissue morphology related to BHDQ. Animals with CCl _4_-induced liver injuries treated with BHDQ had less oxidative stress compared to animals with CCl _4_-induced liver injury. BHDQ promoted activation changes in superoxide dismutase, catalase, glutathione peroxidase, glutathione reductase, and glutathione transferase on control values in animals with CCl _4_-induced liver injury. BHDQ also activated gene transcription in *Sod1* and *Gpx1* *via* nuclear factor erythroid 2-related factor 2 and forkhead box protein O1 factors. Therefore, the compound of concern has a hepatoprotective effect by inhibiting the development of necrotic processes in the liver tissue, through antioxidation.

## Introduction

Liver disease remains one of the ten leading causes of death worldwide ^[ 1] ^. When the liver is exposed to toxins, including carbon tetrachloride (CCl _4_), metabolic dysfunction can occur, causing liver fibrosis, cirrhosis, or hepatocellular carcinoma in some instances ^[ 2] ^. CCl _4_ injections are thought to generate reactive molecules with cytochrome P450 2E1 (Cyp2E1) and through trichloromethyl peroxyl radical formation ^[ 3] ^. The resulting oxidative stress can promote lipid peroxidation and the damage of hepatocellular membrane, release of proinflammatory chemokines and cytokines and development of inflammation and apoptosis ^[ 4] ^. Therefore, protecting the liver from oxidative stress is necessary and can be initiated with antioxidant enzymes superoxide dismutase (SOD) and catalase. Likewise, glutathione peroxidase (GP) can oxidize glutathione (GSH) which reduces levels of organic and inorganic peroxides. The reduction of oxidized glutathione (GSSG) can also be catalysed by glutathione reductase (GR). This means that glutathione S-transferases (GSTs) found in liver cells, can be considered the main group of protective enzymes against chemical stress caused by toxins and carcinogenesis ^[ 5] ^.

Despite the large number of hepatoprotective agents, they all have some disadvantages. For example, silymarin loses its importance in acute liver damage due to low bioavailability and the need for long-term use by patients ^[ 6] ^. Unfortunately, silybin is not particularly water soluble, and is not easily absorbed through the intestines ^[ 7] ^. Similarly, ursodeoxycholic acid which can be used as an intervention has also been associated with difficulties similar to those encountered with silybin ^[ 7] ^. An alternative is sulpho-adenosylmethionine which has been trialled and appears to cause anxiety and diarrhoea in some patients ^[ 8] ^. So, despite having relatively manageable side effects these interventions are not considered appropriate. Vitamin E is another potential alternative although there are no compositional standards and the pro-oxidant effects caused by high concentrations are concerns ^[ 9] ^. Finally, selonsertib has been shown to reduce the degree of bridging fibrosis and cirrhosis, but is again associated with significant adverse events ^[ 9] ^. Therefore, we know which mechanisms target to improve outcomes but we do not yet have a safe and effective intervention.

**Figure 1:**
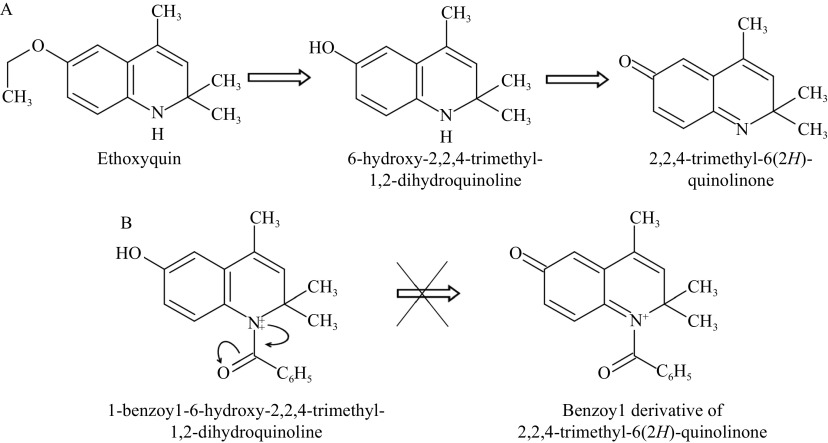
Ethoxyquin metabolism and structure of BHDQ.

Given the central role of oxidative stress in the pathogenesis of toxic liver damage, research into the hepatoprotective effect of substances with high antioxidant potential would seem necessary. Compounds including synthetic antioxidant ethoxyquin have not been approved for use in humans. In a previous study, we demonstrated the hepatoprotectivity of a derivative, 6-hydroxy-2,2,4-trimethyl-1,2-dihydroquinoline ^[ 10] ^. After conducting that study, we inserted a benzoyl group into the structure of this compound and created a new derivative, 1-benzoyl-6-hydroxy-2,2,4-trimethyl-1,2-dihydroquinoline (BDHQ). BHDQ has the potential to prevent the formation of a toxic 2,2,4-trimethyl-6(2 *H*)-quinolinone from the compound ( ***Fig. 1***) ^[ 11] ^.

Therefore, we assessed the antioxidization and hepatoprotective properties of BHDQ in rodents with acute liver damage. Here, we tested a dihydroquinoline derivative in terms of hepatocyte cytolysis markers and histopathologic changes in the liver. We also looked to describe how BHDQ inhibits the development of oxidative stress and modulates antioxidation in rats with CCl _4_-induced liver damage. Finally, we compared silymarin to BHDQ in terms of hepatoprotectivity and antioxidization.

## Materials and methods

### Reagents

BHDQ was synthesized according to a previously developed scheme ^[ 11] ^. The LD50 of the compound was 800 mg/kg body weight (BW). Diagnostic kits B 01.01 ALT-VITAL, B 02.01 AST-VITAL and B 17.11 Triglycerides-Vital were obtained from Vital Development Corporation (Russia). Diagnostic kit 007.004 Gamma-GT-Olvex was purchased from Olvex Diagnosticum (Russia). 1-chloro-2,4-dinitrobenzene acid and 5,5′-dithiobis (2-nitrobenzoic acid) were obtained from Sigma Aldrich (USA). Citrate was purchased from PanReac (Spain). GSH, NADPH and nitroblue tetrazolium (NBT) were from AppliChem (Germany). All products related to polymerase chain reaction (PCR) were from Eurogen (Russia). Caspase-8 and caspase-3 kits were obtained from Abcam (UK). BCA Protein Assay Kit Ⅱ was obtained from BioVision (USA).

### Animals

Sixty male Wistar rats, 4–6 months old and weighing 200–250 g, were housed under normal light-dark conditions (12 hours light followed by 12 hours dark) for the entire experiment and had access to food and water, *ad libitum*. The food comprises the ingredients of: barley, oats, wheat bran, meat and bone meal, table salt, and lime flour. The nutrient content of the laboratory animal diets was shown in ***Supplementary Table 1*** (available online).

The study protocol was approved by the Ethics committee on biomedical research expertise in Voronezh State University (Voronezh, Russia) and was conducted in accordance with the EU directive 2010/63/EU for animal experiments.

**Figure 2:**
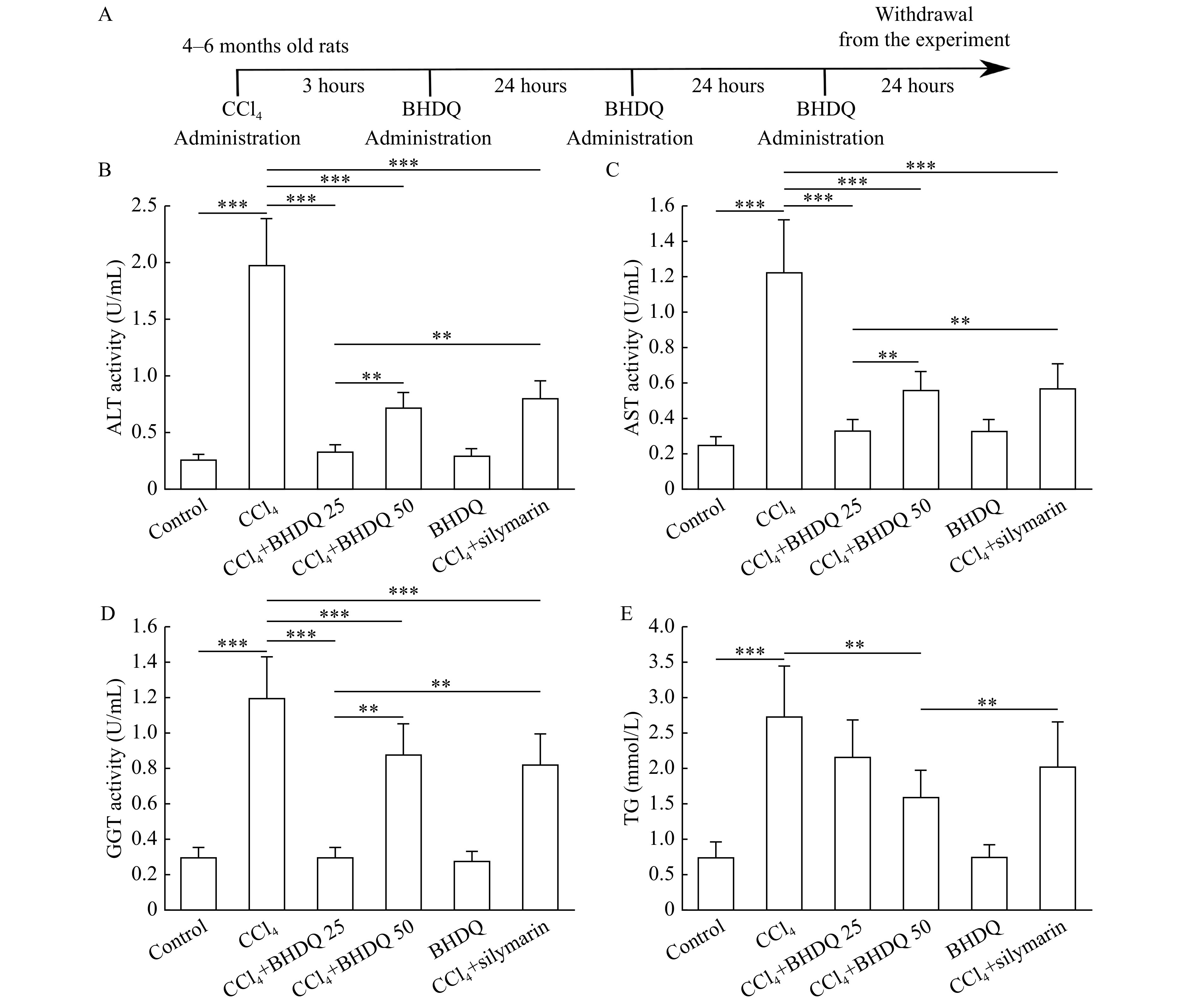
BHDQ reduced the level of marker enzymes of hepatocyte cytolysis in rats with CCl _4_-induced liver injury.

The animals were randomly assigned to either a control, CCl _4_, CCl _4_+BHDQ 25, CCl _4_+BHDQ 50, BHDQ, or a CCl _4_+silymarin group. Each group consisted of 10 rodent subjects. The control group received a single dose of 1 mL of vaseline oil *via* oral gavage. Rats in the CCl _4_ group received a single intragastric dose of CCl _4_ (64 μL dissolved in 1 mL of vaseline oil per 100 g BW) ^[ 10] ^. Rats in CCl _4_+BHDQ 25 and CCl _4_+BHDQ 50 groups received BHDQ (25 and 50 mg respectively, dissolved in 1 mL of 1% soluble starch solution per 1 kg BW) 3 hours after the administration of CCl _4_ and was administrated every 24 hours for three days ( ***Fig. 2A***). Rats in the CCl _4_+silymarin group were treated with 50 mg/kg BW silymarin (Carsil, Sopharma, Bulgaria) after the administration of CCl _4_ under the same scheme. Rats in the BHDQ group were treated with BHDQ (50 mg/kg BW) once per day for three days. The dosage of BHDQ was calculated according to the dosage of Carsil given in the instructions for use. In accordance with the instructions, Carsil is administered for 90 days at a dose of 105–210 mg per day, corresponding to 135 to 270 mg/kg of the drug per 70-kg person. Accordingly, in the CCl _4_+BHDQ 50 and BHDQ groups, animals received a total of 150 mg/kg BW BHDQ over the entire experimental period.

All animals were humanely euthanized on day 4 to harvest blood and liver tissues. On the fourth day from the beginning of the experiment, rats were sacrificed, liver and blood were extracted and immediately used for biochemical analysis.

Liver homogenate and blood serum were used in further studies. Parts of the liver of each rat were frozen at −80 °C for PCR analysis. Each investigated indicator in the samples was analyzed in triplicate.

### Histological staining

Hematoxylin and eosin (H&E) staining of liver was assessed for three rats from each group. Rats were anesthetized with 5 mL chloroform in a soaked cotton bud, in the vacuum sealed glass chamber for 2 minutes ^[ 12] ^. Livers were rapidly removed and immersed in 10% formalin for 2 hours, then washed three times using PBS. After being dehydrated and embedded with paraffin, liver tissues were sliced into 6-µm-thick coronal sections using a rotary microtome HM-325 (Thermo Fisher Scientific, USA) for the H&E staining. High magnification images were captured using an AxioLab A1 light microscope (Zeiss, Germany). A minimum of five fields for each slide were assessed. A numerical scoring for assessing histological activity was carried out in accordance with the approach reported by Knodell *et al* ^[ 13] ^.

### Biochemiluminescence

Oxidative stress intensity and the total antioxidant activity in liver and blood serum were measured through biochemiluminescence (BChL), induced by hydrogen peroxide with iron sulfate ^[ 14] ^. This method is based on the decomposition of H _2_O _2_ by transition valence metal ions (Fe ^2+^) in the Fenton reaction. Generating reactive oxygen species (ROS) initiates oxidation in samples. Recombining formed radicals generate unstable tetroxide, which in turn decomposes with the emission of a quantum of light.

The reaction medium contained 0.4 mL of 0.02 mmol/L potassium phosphate buffer (pH 7.5), 0.4 mL of 0.01 mmol/L FeSO _4_ and 0.2 mmol/L of a 2% H _2_O _2_ solution introduced immediately before measurement. The test sample was added in 0.1 mL amounts before adding the 2% H _2_O _2_ solution. The BChL kinetic curve was recorded for 30 seconds using a BChL-07 biochemiluminometer (Medozons, Russia). The following parameters were determined: the light sum of chemiluminescence (S) and maximum intensity ( *I*_max_) was used to characterize the level of oxidative stress, and tangent of the BChL kinetic curve slope (tgα _2_) was used to characterize characterizing antioxidant activity.

### Biochemical analysis

The activities of alanine aminotransferase (ALT), aspartate aminotransferase (AST), gamma-glutamyl transpeptidase (GGT) and concentration of triglycerides (TG) were measured using diagnostic kits listed in reagents. Concentrations of diene conjugates (DC), citrate and GSH were analyzed as described earlier ^[ 11] ^.

Aconitate hydratase (AH) activity was determined spectrophotometrically at 235 nm. The medium for the AH activity assay included: 50 mmol/L Tris-HCl buffer, pH 7.8, 4 mmol/L citrate ^[ 15] ^. Reactions were initiated by introducing the test sample to a spectrophotometric medium.

SOD activity was measured at 540 nm based on NBT reduction ^[ 16] ^. Catalase activity was assessed according to the method developed by Góth ^[ 17] ^. GP activity was assayed according to Paglia and Valentina ^[ 18] ^. GR activity was assessed by measuring the oxidation of NADPH (0.16 mmol/L) using oxidized glutathione as a substrate ^[ 19] ^. GT activity was assessed using GSH and 1-chloro-2,4-dinitrobenzene acid as substrates ^[ 20] ^. Glucose-6-phosphate dehydrogenase (G6PD) and NADP-isocitrate dehydrogenase (NADP-IDH) activity was measured as described previously ^[ 11] ^.

Enzyme activities of caspase-8 and caspase-3 were measured using colorimetric assay kits according to manufacturer's protocols. The enzymatic activity was evaluated using a Hitachi U1900 spectrophotometer (Japan).

### Quantitative reverse transcription PCR

Total RNA was isolated from liver tissue using ExtractRNA reagent (Eurogen, Russia). Quantitative reverse transcription PCR was performed using qPCRmix-HS SYBR with a BioRad Connect device (BioRad, USA) according to the manufacture's instruction. The mRNA level of each gene was normalized to house-keeping genes such as *Gapdh* and *Actb*. Primers were listed in ***Supplementary Table 2*** (available online).

### Statistical analysis

Multiple groups were analyzed using a one-way ANOVA with Tukey's *post hoc* test, *P*<0.05 was considered statistically significant. Statistical analysis was performed using the IBM SPSS Statistics (version 25.0) software. All quantitative data are presented as mean±SD.

## Results

### BHDQ reduced pathological changes in CCl _4_-induced liver injury in rats

We found that CCl _4_-induced hepatic injury was associated with an increase in ALT ( *P*<0.001), AST ( *P*<0.001), and GGT ( *P*<0.001) activity in blood serum of animals compared to the control group. There was also an increase in TG concentration in the liver of animals with CCl _4_-induced hepatic injury compared to the control group ( *P*<0.001). At the same time, the administration of BHDQ with CCl _4_-induced hepatic injury decreased the level of ALT ( *P*<0.001), AST ( *P*<0.001), and GGT ( *P*<0.001) compared to the CCl _4_ group, and the dose of BHDQ of 25 mg/kg BW appeared more effective ( *P*=0.002 for ALT and AST, *P*=0.001 for GGT) ( ***Fig. 2B***– ***E***).

Silymarin did not differ from that of BHDQ in terms of efficacy, in relation to liver functions, at a dose of 50 mg/kg BW. However, the effectiveness of silymarin was lower than BHDQ, at a dose of 25 mg/kg BW ( *P*=0.003 for ALT and AST, *P*=0.001 for GGT). There was also a significant reduction ( *P*=0.001) in TG concentrations which appears to have been facilitated by administering the BHDQ at a dose of 50 mg/kg BW, compared to the CCl _4_ group ( ***Fig. 2E***).

**Figure 3:**
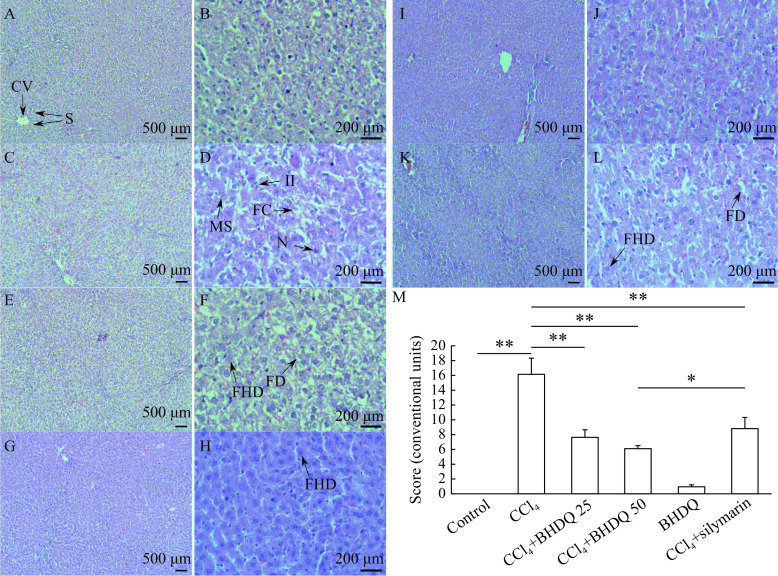
BHDQ reduced histopathological changes in the liver of rats treated with CCl _4_.

Histological analysis of liver tissue morphologies confirmed the protective effect of BHDQ in CCl _4_-induced hepatic injury. The liver in the control group showed normal hepatocytes, obvious sinusoids (S), and central vein (CV) ( ***Fig. 3A*** and ***B***). Liver tissues in the CCl _4_ group was characterized by focal necrotic cell death (N), diffuse fatty changes (FC), microvesicular steatosis in hepatocyte cytosol (MS), and inflammatory infiltration (II) ( ***Fig. 3C*** and ***D***). Livers in the CCl _4_+BDHQ 25 ( ***Fig. 3E*** and ***F***) and CCl _4_+silymarin ( ***Fig. 3K*** and ***L***) groups showed less severe liver injury with some pathological fatty deposition (FD) and focal hepatocellular degeneration (FHD). No fatty liver dysplasia was observed in the CCl _4_+BDHQ 50 ( ***Fig. 3G*** and ***H***) group. BDHQ group indicated no pathologic lesions ( ***Fig. 3I*** and ***J***). A numerical scoring for histopathological changes is present in ***Fig. 3M***. Therefore, BHDQ showed a hepatoprotective effect in CCl _4_-induced liver damage and was more effective than silymarin in relation to activity of ALT, AST, GGT, concentration of TG and histopathological score ( *P*<0.05).

### BHDQ improved the redox status in the liver and serum with CCl _4_-induced liver damage

In order to understand the role of oxidative stress in the pathogenesis of toxic liver damage, we analyzed the effect of BHDQ on redox status in rats treated with CCl _4_. CCl _4_ appeared to cause an increase in *I*_max_ ( *P*<0.001) and S ( *P*<0.001), reflecting the intensity of free radical oxidation in the liver and blood serum, compared to animals in the control group ( ***Table 1*** and ***Table 2***).

**Table 1:** Redox status in the liver of experimental rats

Indicator	Control	CCl _4_	CCl _4_+BHDQ 25	CCl _4_+BHDQ 50	BHDQ	CCl _4_+silymarin
*I*_max_ (mV)	42.03±8.41	86.72±17.29 ^***^	44.02±6.21 ^##^	39.04±5.37 ^###^	33.62±6.72 ^*^	63.58±12.71 ^##,&&^
S (mV*s)	92.32±4.61	236.04±11.82 ^***^	120.88±24.23 ^##^	98.71±19.69 ^###^	91.37±18.32	112.06±22.41 ^##^
tgα _2_	8.11±1.60	16.70±3.31 ^***^	10.22±2.28 ^##^	8.91±1.80 ^###^	6.51±1.33	14.11±2.80 ^#,&&^
DC (µmol/mL homogenate)	9.01±1.80	15.71±3.14 ^***^	8.47±1.72 ^###^	10.20±2.00 ^###^	9.91±2.02	13.03±2.63 ^##,&^
AH (U/g tissue)	0.249±0.062	0.083±0.021 ^***^	0.177±0.043 ^##,&^	0.207±0.052 ^###^	0.224±0.056	0.150±0.038 ^###,&&^
Citrate (µmol/mL homogenate)	0.65±0.13	1.31±0.26 ^***^	0.84±0.17 ^##,&^	0.64±0.13 ^###^	0.70±0.14	0.75±0.15 ^##,&&^
Data are presented as mean±SD ( *n*=10 in each group). Statistical analysis was performed using one-way ANOVA with Tukey's *post hoc* test. ^*^ *P*<0.05 and ^***^ *P*<0.001 *vs.* the control group; ^#^ *P*<0.05, ^##^ *P*<0.01, and ^###^ *P*<0.001 *vs.* the CCl _4_ group; ^&^ *P*<0.05 and ^&&^ *P*<0.01 *vs.* the CCl _4_+BHDQ 50 group. *I*_max_: maximum intensity of chemiluminescence; S: light sum of chemiluminescence; tgα _2_: tangent of the biochemiluminescence kinetic curve slope; DC: diene conjugates; AH: aconitate hydratase.						

**Table 2:** Redox status in blood serum of experimental rats

Indicator	Control	CCl _4_	CCl _4_+BHDQ 25	CCl _4_+BHDQ 50	BHDQ	CCl _4_+silymarin
*I*_max_ (mV)	25.22±6.31	51.20±12.78 ^***^	35.10±8.83 ^###,&^	21.71±5.40 ^###^	23.56±5.87	28.00±7.03 ^###,&^
S (mV*s)	307.70±76.91	739.79±184.92 ^***^	305.04±76.30 ^###^	317.22±79.31 ^###^	292.33±73.08	314.11±78.52 ^###^
tgα _2_	13.30±2.72	29.51±5.93 ^***^	15.04±2.76 ^###^	13.41±3.41 ^###^	11.31±2.3	15.40±3.13 ^###,&^
DC (µmol/mL)	7.50±1.51	16.04±3.22 ^***^	8.95±1.81 ^###^	10.42±2.11 ^###^	7.13±1.41	9.90±2.00 ^###^
AH (U/mL)	0.061±0.012	0.025±0.005 ^***^	0.029±0.003 ^&^	0.061±0.015 ^###^	0.067±0.013	0.061±0.012 ^###^
Citrate (mmol/L)	0.57±0.11	1.24±0.25 ^***^	1.17±0.23 ^&^	0.61±0.11 ^###^	0.54±0.10	0.77±0.15 ^##,&^
Data are presented as mean±SD ( *n*=10 in each group). Statistical analysis was performed using one-way ANOVA with Tukey's *post hoc* test. ^***^ *P*<0.001 *vs.* the control group; ^##^ *P*<0.01 and ^###^ *P*<0.001 *vs.* the CCl _4_ group; ^&^ *P*<0.05 *vs.* the CCl _4_+BHDQ 50 group. *I*_max_: maximum intensity of chemiluminescence; S: light sum of chemiluminescence; tgα _2_: tangent of the biochemiluminescence kinetic curve slope; DC: diene conjugates; AH: aconitate hydratase.						

Total antioxidant activity in the liver and serum also increased which was evidenced by an increase in tgα _2_ ( *P*<0.001) ( ***Table 1*** and ***Table 2***). In addition, rats with CCl _4_-induced liver damage were characterized by an increased level of primary products of lipid peroxidation ( *i.e.*, DC) ( *P*<0.001), decreased activity of AH ( *P*<0.001), sensitive to ROS, and accumulation of AH substrate citrate ( *P*<0.001), compared to the control group. BHDQ at a dose of 50 mg/kg BW changed these parameters moving them closer to control values in contrast to animals of CCl _4_ group (all *P*<0.001), but strong evidence of greater efficacy for all dosages was not obtained ( ***Table 1*** and ***Table 2***). Data indicate that BHDQ exhibited hepatoprotective activity due to the presence of an antioxidant effect.

Silymarin also contributed to a significant change in all analyzed parameters in rats with CCl _4_-induced liver damage (all *P*<0.001). However, this was less effective in relation to *I*_max_, tgα _2_ and AH activity, DC concentration in the liver, and citrate level compared to BHDQ at a dose of 50 mg/kg BW ( *P*<0.001 for *I*_max_, *P*=0.001 for tgα _2_, *P*=0.009 for AH activity, *P*=0.01 for DC concentration, and *P*=0.001 for citrate level) ( ***Table 1*** and ***Table 2***).

### BHDQ modulated antioxidant enzyme activation and NADPH-supplying enzymes in CCl _4_-induced liver injury

**Figure 4:**
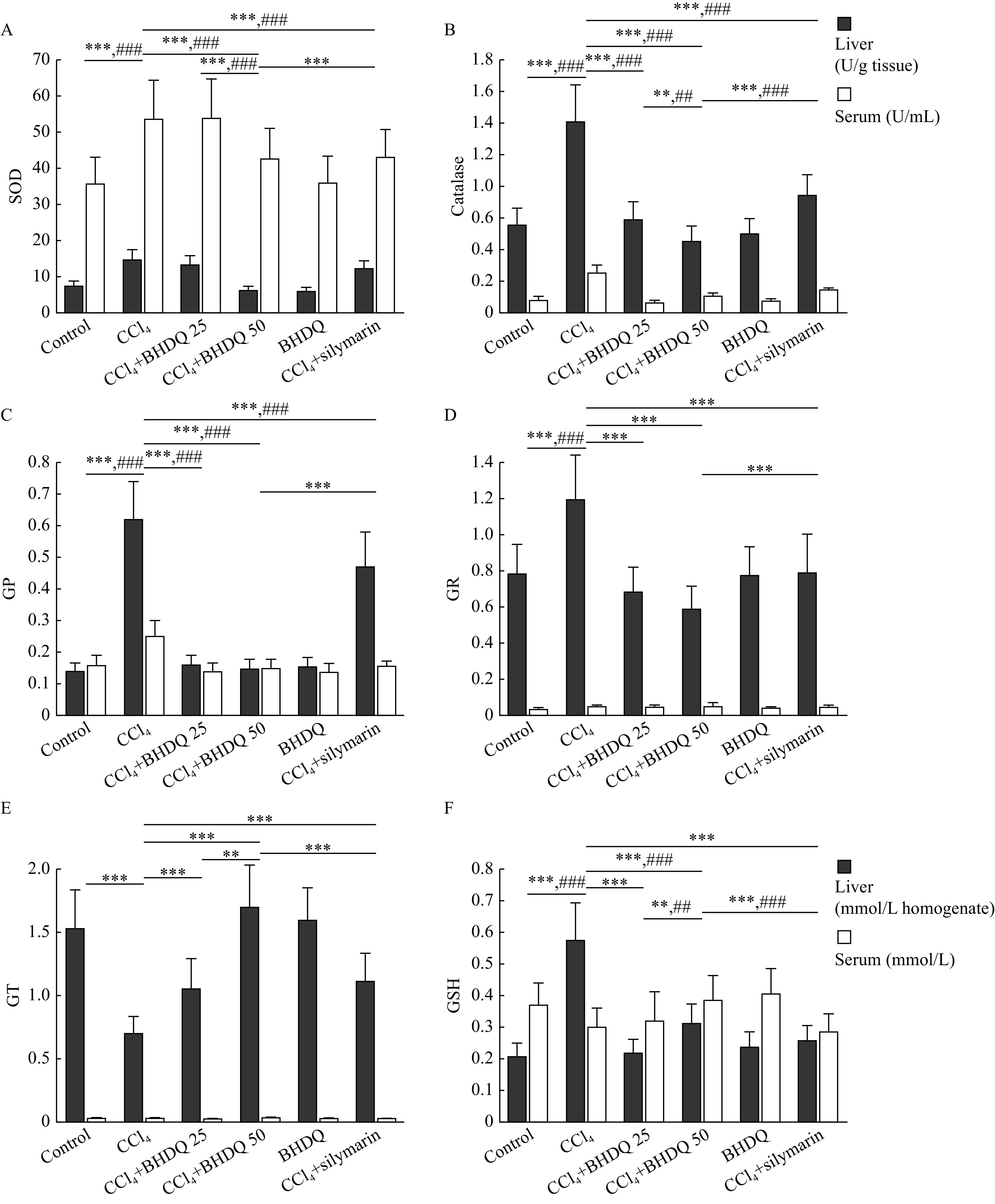
BHDQ promoted normalization of antioxidant enzymes activity in CCl _4_-induced liver damage.

Protection against ROS formed during CCl _4_ metabolism is provided by an antioxidant system including enzymes such as SOD, catalase, GP, GR, and GT. The administration of CCl _4_ to laboratory animals was associated with a significant (all *P*<0.001) increase in the activity of SOD, catalase, GP and GR in the liver and blood serum compared to the control group ( ***Fig. 4A***– ***D***). A decrease in GT activity in the liver ( *P*<0.001) and a multi-directional change in the content of GSH in liver and blood serum (all *P*<0.001) were observed in animals with CCl _4_ group ( ***Fig. 4E*** and ***F***). In turn, BHDQ at a dose of 50 mg/kg BW contributed to a change in the activity of SOD, catalase, GP, GR, GT and GSH concentration on control values (all *P*<0.001) in rats with CCl _4_-induced liver injury.

For the activity of SOD ( *P*<0.001), catalase ( *P*=0.001), GT ( *P*=0.001) and GSH concentration ( *P*=0.003), more significant changes relative to BHDQ 25 group were observed for BHDQ at a dose of 50 mg/kg BW ( ***Fig. 4A***– ***F***). Additionally, the effectiveness of silymarin was significantly lower (all *P*<0.001) based on the analysis of SOD, catalase, GP, GR, GT activity and GSH concentration compared with the CCl _4_+BHDQ 50 group, or with both groups of rodents with CCl _4_-induced liver injury receiving BHDQ ( ***Fig. 4A***– ***F***).

**Figure 5:**
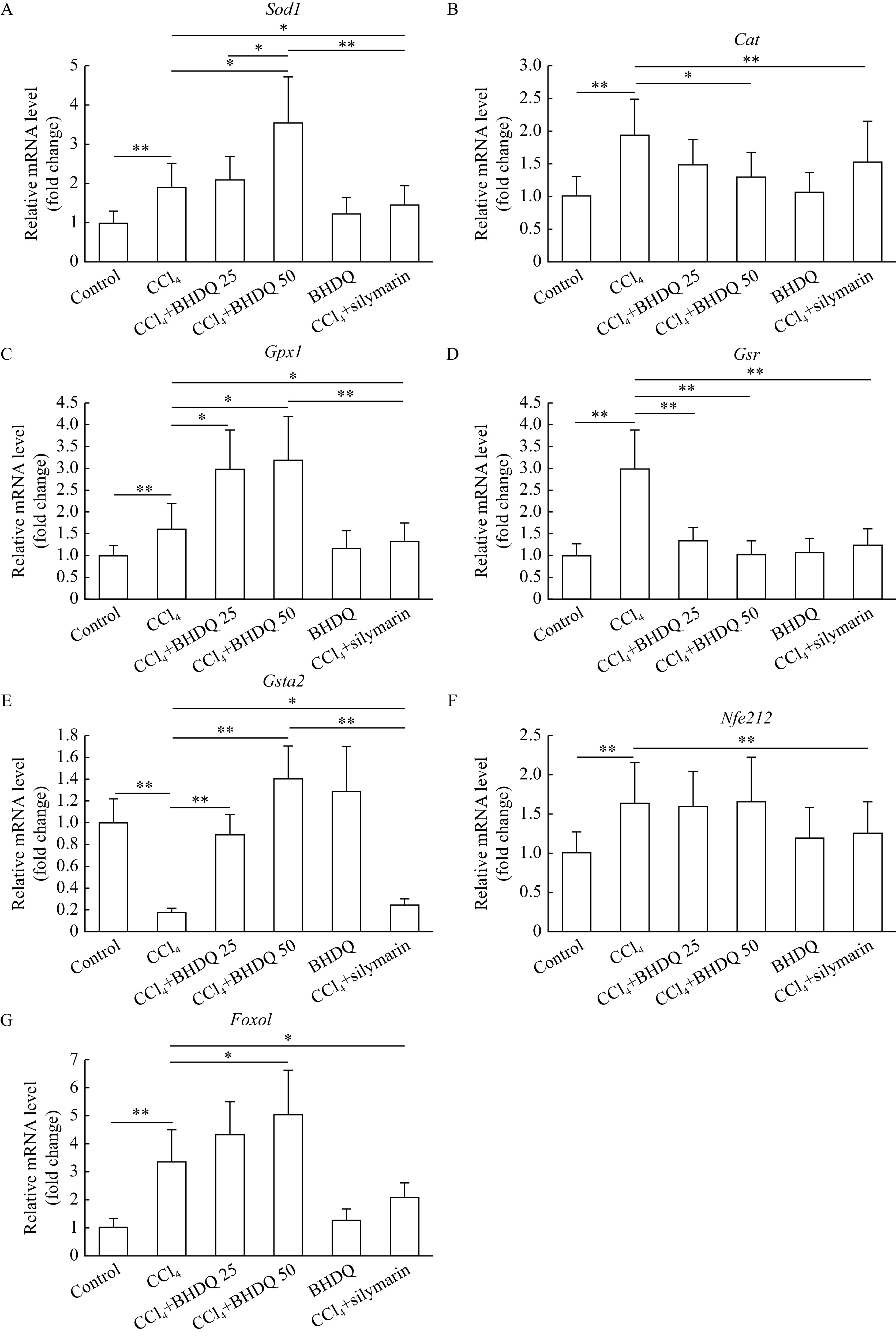
BHDQ had a modulating effect on mRNA levels of antioxidant response genes of rats treated with CCl _4_.

The induction of toxic hepatic injury in rats was associated with activated gene expression of *Sod1* ( *P*=0.001), *Cat* ( *P*=0.001), *Gpx1* ( *P*=0.009), *Gsr* ( *P*=0.001), *Nfe2l2* ( *P*=0.002) and *Foxo1* ( *P*=0.001) ( ***Fig. 5A***– ***D***, ***F*** and ***G***) compared to control group; however, a decrease in the level of mRNA of the *Gsta2* ( *P*<0.001) gene was observed ( ***Fig. 5E***). BHDQ at a dose of 50 mg/kg BW for rats with CCl _4_-induced hepatic injury was associated with additional activation of expression of *Sod1* ( *P*=0.01), *Gpx1* ( *P*=0.011) and *Foxo1* ( *P*=0.021) ( ***Fig. 5A***, ***C*** and ***G***). The level of *Cat* ( *P*=0.036), *Gsr* ( *P*=0.002) and *Gsta2* ( *P*=0.003) transcripts changed toward control when BHDQ was administered to animals of CCl _4_ group ( ***Fig. 5B***, ***D*** and ***E***). Whereas, silymarin changed in terms of *Sod1*, *Cat*, *Gpx1*, *Gsr*, *Gsta2*, *Nfe2l2* and *Foxo1* expression on values of control group ( *P*<0.05).

BHDQ had a more significant modulatory effect on the functioning of the antioxidant system compared with silymarin.

**Figure 6:**
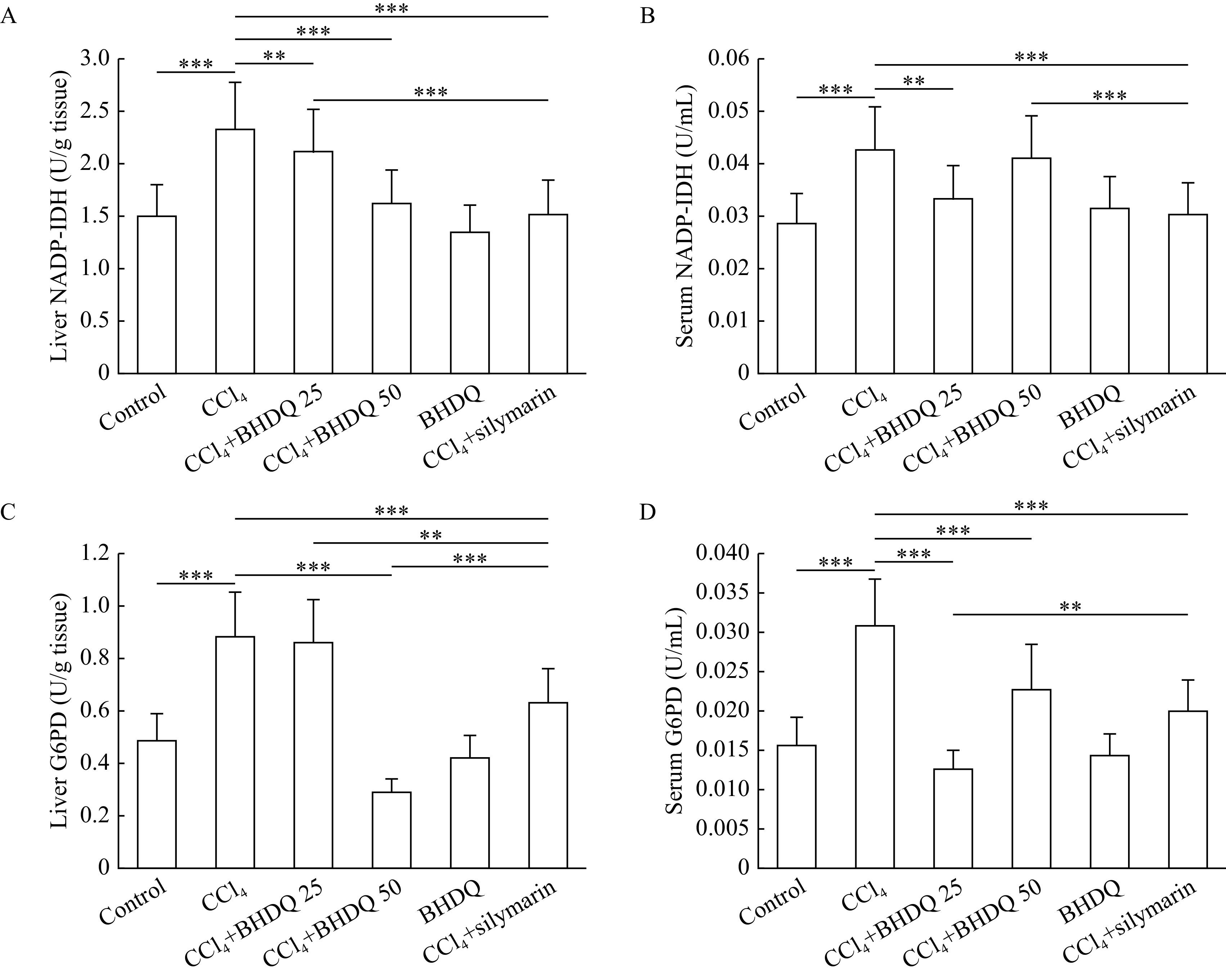
The activity of NADPH-generating enzymes in rats with CCl _4_-induced liver injury changed on values of control group under the action of BHDQ.

It was found that CCl _4_-induced hepatic injury in rats was associated with NADP-IDH ( ***Fig. 6A*** and ***B***) and G6PD ( ***Fig. 6C*** and ***D***) activation in the liver and blood serum compared to the control group (all *P*<0.001). BHDQ at a dose of 50 mg/kg BW contributed to the change of NADP-IDH and G6PD activity on the control values in liver of rats with CCl _4_-induced hepatic injury (all *P*<0.001). However, similar dose-dependencies were not observed when analyzing tissues. There was no unequivocal evidence for a more effective effect of both doses of BHDQ compared to silymarin on NADPH-supplying enzymes.

### BHDQ had no significant effect on caspase activity in CCl _4_-induced liver injured rats

**Figure 7:**
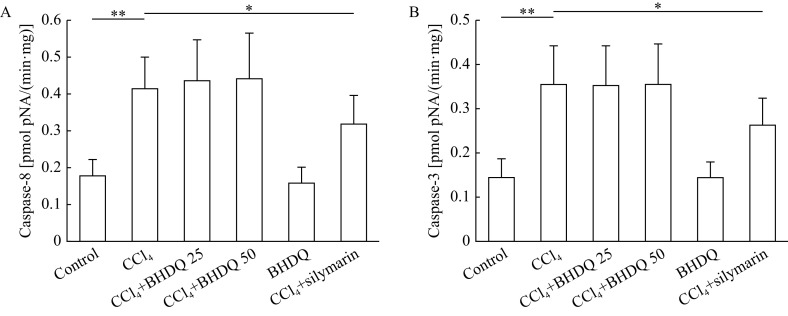
BHDQ had no significant effect on caspase activity in CCl _4_-induced liver injury.

Oxidative stress and inflammation developing through CCl _4_-induced liver injury can initiate apoptosis by activating inducible and effector caspases. As this study has shown, CCl _4_-induced hepatic injury promoted the activation of inductor caspase-8 ( *P*=0.001) ( ***Fig. 7A***) and effector caspase-3 ( *P*=0.001) compared to the values of the control animals ( ***Fig. 7B***) ( *P*<0.05). However, BHDQ did not significantly affect caspases activities while silymarin did (all *P*<0.05).

## Discussion

Oxidative stress impairs hepatic parenchyma cells, extracellular matrix, and immune activation, thus playing a central role in CCl _4_-induced hepatic injury ^[ 4] ^. It has previously been shown that the pronounced antioxidant effect of 6-hydroxy-2,2,4-trimethyl-1,2-dihydroquinoline reduces CCl _4_-induced hepatic injury ^[ 10] ^. However, it is also known that ethoxyquin, an antioxidant from the same series of compounds, has pro-oxidant as well as carcinogenic effects ^[ 21] ^. Here we structurally modified 6-hydroxy-2,2,4-trimethyl-1,2-dihydroquinoline by introducing a benzoyl group to the nitrogen atom. This was done to prevent the generation of the toxic metabolite 2,2,4-trimethyl-6(2 *H*)-quinolinone ^[ 11] ^.

We found in terms of hepatoprotectivity that BHDQ was related to a decreased level of oxidative stress, marker enzymes of hepatic cytolysis. We also observed improved morphologies in the liver tissue in rats treated with CCl _4_. Increased ROS production induced by CCl _4_ is known to play a role in the development of hepatic steatosis. Therefore, activation of Cyp2E1 by CCl _4_ leads to the formation of toxic peroxyl and alkoxyl radicals which initiate lipid peroxidation ^[ 22] ^. In turn, BHDQ reduced fatty liver infiltration and probably, BHDQ antioxidation became the key mechanism involved in hepatoprotectivity in regard to this compound. Thus, the reduction of oxidative stress and fatty infiltration of hepatocytes under the action of BHDQ appeared to be the key factor in the improvement of histopathological score of liver parenchyma and the reduction of hepatic enzyme activity in serum of rats with CCl _4_-induced hepatic injury.

The liver has its own antioxidant defence system against ROS. The main antioxidant enzymes include SOD, catalase, GP, GT, and GR, which catalyze the reduction of GSSG to GSH. NADPH is required for the reduction of GSSG in the GR-catalysed reaction which essentially constrains the limiting factor. The main suppliers of NADPH are NADP-IDH and G6PD ^[ 23] ^. We demonstrated an increase in the activity of the majority of the antioxidant enzymes in the CCl _4_ group. This was apparently the result of an adaptive response to the introduction of xenobiotics and the development of oxidative stress.

Along with this, CCl _4_ was associated with an imbalance in the antioxidant system, which was expressed as inhibited GT activity and in multidirectional changes related to GSH concentrations. BHDQ in rats with CCl _4_-induced liver damage contributed to the normalization of the analyzed. BHDQ appeared to exhibit antioxidant activity and improved the redox status in the liver of animals with CCl _4_-induced liver damage. Thus, the change in the activity of antioxidant enzymes could be the result of a decrease in their load during the reduction of ROS level by BHDQ.

One of the central regulators of the antioxidant system is the Nrf2 transcription factor which is encoded by the *Nfe2l2* gene ^[ 24] ^. Oxidative stress also causes a significant increase in the transcriptional activity of Foxo1, and this affects the expression of SOD and catalase ^[ 25] ^. Our findings revealed an increase in mRNA levels of antioxidant enzyme genes in CCl _4_-dependent hepatic injury. However, a decrease in expression of *Gsta2*, which correlated with GT activity in the liver, was also revealed. BHDQ administration contributed to a decrease in *Cat* and *Gsr* transcript levels, and an increase in *Gsta2* mRNA levels, relative to animals with CCl _4_-induced hepatic injury. We also found that there was additional activation of the *Sod1, Gpx1* and *Foxo1* genes in this study.

BHDQ appears to have a stimulating effect on the expression of genes encoding antioxidant enzymes. It can be assumed that BHDQ effect manifests by way of *Nfe2l2* and *Foxo1* and by transcriptional regulation. In particular, it is known that dihydroquinoline ethoxyquin is a selective activator of Nrf2 ^[ 26] ^. Therefore we suggest, that despite the decrease in oxidative stress intensity and the normalization of antioxidant enzymes, the content of mRNA encoding for these enzymes still increased with BHDQ. This somewhat counter-intuitive idea is also associated with the apparent inducing effect which should be considered more closely. It appears that the normalization of SOD and GP activity could be related to other regulatory mechanisms, in particular a change in their catalytic properties.

In CCl _4_-induced damage, ROS attack various hepatocellular structures causing the release of proinflammatory mediators. In particular, tumor necrosis factor-α (TNF-α) is released, which contributes to the development of apoptosis. TNF-α activates the Fas ligand, which is followed by the generation of an apoptotic complex for binding and activation of procaspase-8. Caspase-8 can then activate effector caspase-3, which ultimately leads to cellular apoptosis ^[ 27] ^. In this study, we noticed an increase in caspase-8 and caspase-3 activity after CCl _4_. BHDQ had no effect on the activity of these apoptosis mediating enzymes. It is likely that the limiting factor was duration of the experiment which was four days from CCl _4_ administration. This was most likely insufficient to track the effect of BHDQ on caspase activity.

In summary, we did not observe BHDQ dose-dependency in relation to oxidative stress or other indicators. There was more pronounced hepatoprotectivity in relation to this compound at a dose of 25 mg/kg BW. This activity does appear to be superior to silymarin, although this was a relatively small rodent modelling study. We can conclude that further BHDQ studies are warranted although we must pay particular attention to dose-responses. Providing BHDQ to rats with CCl _4_-induced hepatic injury had a hepatoprotective effect which is associated with the antioxidation capacity of the tested compound.
